# The association between age-related macular degeneration and risk of Parkinson disease: A systematic review and meta-analysis

**DOI:** 10.1097/MD.0000000000040524

**Published:** 2024-11-15

**Authors:** Mingxian Meng, Xiaoming Shen, Yanming Xie, Jiabin Wang, Junhong Liu

**Affiliations:** a Encephalopathy Hospital, The First Affiliated Hospital of Henan University of Traditional Chinese Medicine, Zhengzhou, Henan Province, China; b The First Clinical Medical College, Henan University of Traditional Chinese Medicine, Zhengzhou, Henan Province, China; c Institute of Clinical Basic Medicine, China Academy of Chinese Medical Sciences, Beijing, China.

**Keywords:** age-related macular degeneration, meta-analysis, Parkinson disease

## Abstract

**Background:**

Numerous cohort studies have explored the association between age-related macular degeneration (AMD) and Parkinson disease (PD). However, a comprehensive meta-analysis on this topic is currently lacking. This study aims to address this gap by conducting a meta-analysis of existing cohort studies to investigate the relationship between AMD and the risk of developing PD.

**Methods:**

Relevant studies were systematically identified through thorough searches of the PubMed, Web of Science, Embase, and Cochrane Library databases. Two investigators independently conducted data extraction. Cohort studies meeting the eligibility criteria and providing risk and precision estimates regarding AMD and the risk of PD were included. Pooled hazard ratio (HR) accompanied by 95% confidence interval (CI) were calculated using either a random-effects model or a fixed-effects model. Sensitivity analyses, involving the exclusion of 1 study at a time, were performed to assess the robustness of the findings. Publication bias was evaluated using Egger test.

**Results:**

Five studies were included, encompassing a total of 4,771,416 individuals. Among these, 128,771 individuals had AMD, while 4,642,645 individuals did not. The pooled analysis revealed a significant increase in the risk of developing PD for individuals with age-related macular degeneration (hazard ratio [HR] = 1.44; 95% confidence interval [CI]: 1.22–1.71; *I*^2^ = 47.3%). Sensitivity analysis confirmed the robustness of the results. For the exploration of the relationship between nAMD and the risk of developing PD, 2 cohorts were included. The pooled analysis demonstrated a significantly elevated risk of PD for individuals with nAMD (HR = 2.21; 95% CI: 1.55–3.16; *I*^2^ = 0%).

**Conclusion:**

This meta-analysis suggests a significant association between AMD and an increased risk of PD. These findings offer fresh perspectives on PD’s etiology and pathogenesis, but should be interpreted with caution given the limitations in establishing causality.

## 1. Introduction

Parkinson disease (PD) is an increasingly prevalent neurodegenerative condition, with its global incidence continually rising.^[1]^ It is anticipated that by the year 2040, the global population of individuals affected by Parkinson disease will exceed 12 million.^[2]^ Currently, there are no identified treatments capable of slowing or halting the progression of PD, underscoring the importance of early diagnosis and the identification of risk factors as pivotal strategies for intervention.^[1]^ Published meta-analyses have substantiated that PD is linked to risk factors including air pollution, smoking and pesticide exposure.^[3–5]^ Additionally, research has confirmed an association between visual impairment and an elevated risk of developing PD.^[6]^

Age-related macular degeneration (AMD) emerges as a primary contributor to visual impairment in individuals aged 65 and above.^[7]^ Projections indicate that by the year 2040, the population affected by age-related macular degeneration, characterized by macular degeneration, will surpass 288 million.^[8]^ Optical coherence tomography studies have revealed a corresponding thinning of the retinal nerve fiber layer (RNFL) in patients with PD and AMD.^[9,10]^ Recent literature indicates that iron accumulation, lipid peroxidation, and disruptions in cellular processes are not confined to either AMD or PD alone but may represent key elements linking the 2 conditions.^[11]^ Additionally, both disorders share risk factors such as aging, inflammation, and oxidative stress,^[12]^ providing further indications of a potential association.

Given the strong associations between AMD and PD, our study aims to provide a comprehensive synthesis of the available evidence, which could offer valuable insights for clinical practice. This systematic review and meta-analysis provides an important synthesis of the current evidence regarding the association between AMD and the risk of PD. The findings could have significant implications for clinical practice, especially in the early identification of individuals at higher risk for PD. Understanding the link between AMD and PD may support clinicians in implementing more comprehensive monitoring and management strategies for patients with AMD, potentially leading to earlier interventions aimed at preventing or delaying the onset of PD.

## 2. Methods

This meta-analysis strictly adheres to the PRISMA guidelines for reporting systematic reviews and meta-analyses.^[13]^ Our study protocol has been preregistered on the international meta-analysis and systematic review registration platform PROSPERO under registration number (CRD42023487576).

### 2.1. Ethic statement

All data utilized in this manuscript are derived from publicly accessible databases and do not contain any personal information. Consequently, ethical approval and informed consent procedures are not applicable for this study.

### 2.2. Data sources

The databases searched for this study encompassed PubMed, Web of Science, Embase, and the Cochrane Library, with a cutoff date set at November 22, 2023. The search was not constrained by language, utilizing a combination of medical subject headings (Mesh) and keywords. The search terms comprised (“macular degeneration” OR “age-related macular degeneration” OR “macular degeneration, age”) AND (“Parkinson” OR “Parkinson disease”). Detailed information on the search strategies can be found in Tables S1 to S4, Supplemental Digital Content, http://links.lww.com/MD/N912.

### 2.3. Eligibility criteria

All included studies met the following criteria: cohort studies or case control studies based on cohort trials; the correlation between AMD and the risk of developing PD had to be examined in all studies; detailed descriptions of diagnostic criteria for PD and AMD are available; relative risk (RR)/Hazard Ratio (HR) and corresponding 95% CIs must be provided for calculations.

Studies under the flowing criteria will be excluded: letters, conference abstract, reviews, meta-analysis, duplicated publication; the results could not be calculated; studies without interest outcomes.

### 2.4. Study selection

Two independent reviewers, MMX and SXM, meticulously carried out the literature selection process according to pre-established inclusion and exclusion criteria. Initially, duplicate and irrelevant articles were identified and prioritized for exclusion through a careful examination of titles and abstracts. Subsequently, potentially eligible articles were retrieved in full text, underwent comprehensive review, and were assessed for final inclusion. Any discrepancies or disagreements between the reviewers were resolved through consultation with a third arbitrator, XYM.

### 2.5. Data extraction

The full text of the included studies underwent a comprehensive review, and pertinent data, such as authors, year of publication, study design, and sample characteristics, were initially extracted. A data extraction form was then developed to outline the specific information to be collected, ensuring both consistency and completeness. Two independent reviewers performed the data extraction to guarantee the accuracy of the collected information through mutual validation.^[14]^ In instances where clarification or additional data were required, authors of relevant studies were contacted to provide necessary details.

### 2.6. Risk of bias

In this study, the Newcastle-Ottawa Quality Rating Scale (NOS) served as the tool for assessing the quality of the literature.^[15]^ Two independent reviewers (MMX and SXM) employed the NOS to evaluate each included study, focusing on 3 crucial quality factors related to selection, comparison, and results. The NOS assigns a maximum of 9 stars to each study based on the thoroughness of its design and reporting, with higher star ratings indicative of higher quality. Throughout the evaluation process, any discrepancies between reviewers were resolved through discussion or by introducing third-party reviewers to ensure consistency and reliability in the assessment.

### 2.7. Statistical analysis

Adjusted Hazard Ratio (HR) with corresponding 95% confidence interval (CI) will be employed for evaluating the link between age-related AMD and the risk of PD To gauge heterogeneity, we will utilize the *χ*^2^ test and *I*^2^ values. A fixed-effects model^[16]^ will be applied if *P* > .1 and *I*^2^ ≤ 50%, while a random-effects model^[17]^ will be selected if *I*^2^* *> 50%, indicating substantial heterogeneity. Sensitivity analyses, crucial for ensuring the robustness of findings, involve systematically excluding 1 study at a time and re-running the analysis to confirm the reliability of the overall effect, ensuring that correlation results are not disproportionately influenced by a single study.^[18]^ To assess potential publication bias, we will conduct a visual inspection of funnel plots and employ the Egger test for a statistical assessment.^[19]^ This step aids in identifying and addressing potential publication bias, thereby enhancing confidence in the study results. Due to the intricate nature of AMD and PD, subgroup analyses based on national origin will be conducted. This approach aims to provide a nuanced and detailed exploration of potential variations in associations among distinct subgroups. All statistical analyses will be executed using Stata statistical software, version 14.0.^[20]^

## 3. Results

### 3.1. Study selection

**Figure 1.**
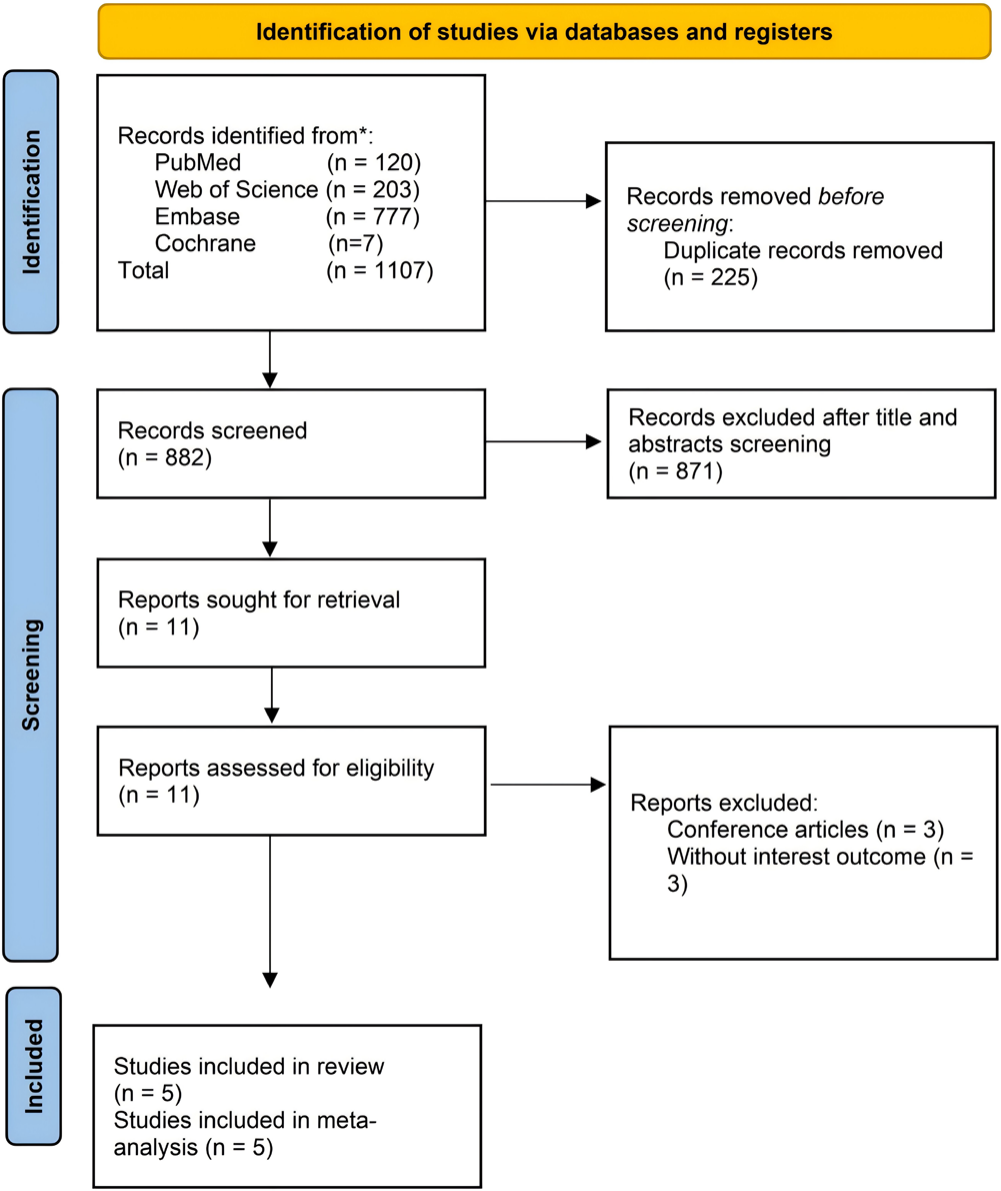
Flow chart for literature screening.

A total of 1107 studies were initially obtained from the database, and after removing 225 duplicates, the remaining 882 studies underwent further screening. Through the evaluation of titles and abstracts, 871 studies were excluded from consideration. Subsequently, the 11 remaining studies underwent a comprehensive review of their full texts, resulting in the inclusion of 5 studies for the meta-analysis.^[12,21–24]^ The entire process of literature screening is visually represented in Figure 1.

### 3.2. Characteristics of included studies

This meta-analysis incorporated data from various studies, with 2^[12,24]^ originated from databases in China, 2^[21,22]^ were sourced from Korean databases, and 1^[23]^ utilizing a Canadian database. Specifically, 2 studies^[23,24]^ focused on investigating the correlation between neovascular age-related macular degeneration (nAMD) and the risk of developing PD. The selected studies for this meta-analysis exclusively adopted retrospective cohort designs, with a minimum follow-up duration of 3 years^[24]^ and a maximum span of 13 years.^[12]^ These studies were published between 2014 and 2023. Notably, the most extensive cohort encompassed 4,151,971 patients with AMD and 53,549 healthy individuals. All studies employed the latest ICD disease codes for the diagnosis of both AMD and PD Crucially.

### 3.3. Quality assessment

All studies included in this analysis achieved Newcastle-Ottawa Scale (NOS) scores exceeding 8 stars, reflecting a high standard of research quality. The NOS scores for each study can be found in Table 1, and comprehensive details are provided in Table S5, Supplemental Digital Content, http://links.lww.com/MD/N912.

**Table 1:** Basic characteristics of the included studies.

Author	Year	Country	Study type	Sample size	Follow-up yr	Age (yr)	Diagnosis of AMD/PD	AMD type	Confounders adjusted	NOS scores
Je Moon Yoon et al^[21]^	2023	South Korea	Retrospective cohort	Total: 4,205,520 AMD: 53,549 No AMD: 4,151,971	10 averages	AMD: 67.36 ± 8.44 No AMD: 60.6 ± 8.31	ICD-10	Age-related macular degeneration	Age, sex, smoking habits, alcohol consumption habits, physical activity, income, place, body mass index, diabetes, hypertension, dyslipidemia and Charlson comorbidity index.	8
Po-Yu Jay Chen et al^[12]^	2021	Taiwan, China	Retrospective cohort	Total: 116,176 AMD: 59,008 No AMD: 57,168	13 averages	AMD: 70.4 ± 9.55 No AMD: 70.3 ± 9.63	ICD-9	Age-related macular degeneration	Sex, age, Comorbidity, Numbers of comorbidity, Medication	9
Seulggie Choi et al^[22]^	2019	South Korea	Retrospective cohort	Total: 308,340 AMD :2213 No AMD: 306,127	8 averages	AMD: 65.9 ± 8.0 No AMD: 60.4 ± 7.8	ICD-10	Age-related macular degeneration	Age, sex, household income, smoking, alcohol consumption, physical activity, body mass index, systolic blood pressure, fasting serum glucose, total cholesterol, and Charlson comorbidity index	9
Mahyar Etminan et al^[23]^	2018	Canada	Retrospective cohort	Total: 131,733 AMD: 13,124 No AMD: 118,609	5 averages	–	ICD-9	Neovascular age-related macular degeneration	Age	8
Shiu-Dong Chung et al^[24]^	2014	Taiwan, China	Retrospective cohort	Total: 9647 AMD: 877 No AMD: 8770	3 averages	66.7 ± 12.3	ICD-9	Neovascular age-related macular degeneration	Age, Sex, Monthly Income, Geographic Region, HTN, DM, HL, And Coronary Heart Disease	9

### 3.4. Meta-analysis of AMD and risk of PD

**Figure 2.**
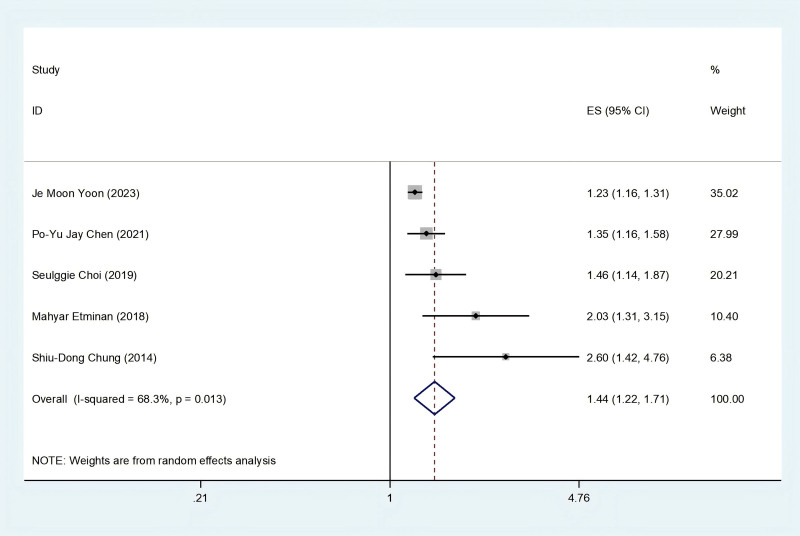
Meta-analysis forest plot of the association between AMD and risk of PD.

Five cohorts^[12,21–24]^ were investigated to explore the correlation between AMD and PD. The findings across all studies consistently indicated a positive association between AMD and the risk of PD. The pooled analysis further confirmed a significant association, revealing an elevated risk of PD in individuals with AMD (HR = 1.44; 95% CI: 1.22–1.71; *I*^2^ = 68.3%, *P *< .001), and the analyses were performed using a random-effects model. Figure 2 illustrates the forest plot depicting the results of the meta-analysis. Sensitivity analysis demonstrated the robustness of these results, as illustrated in Figure S1, Supplemental Digital Content, http://links.lww.com/MD/N912.

### 3.5. Meta-analysis of nAMD and risk of PD

**Figure 3.**
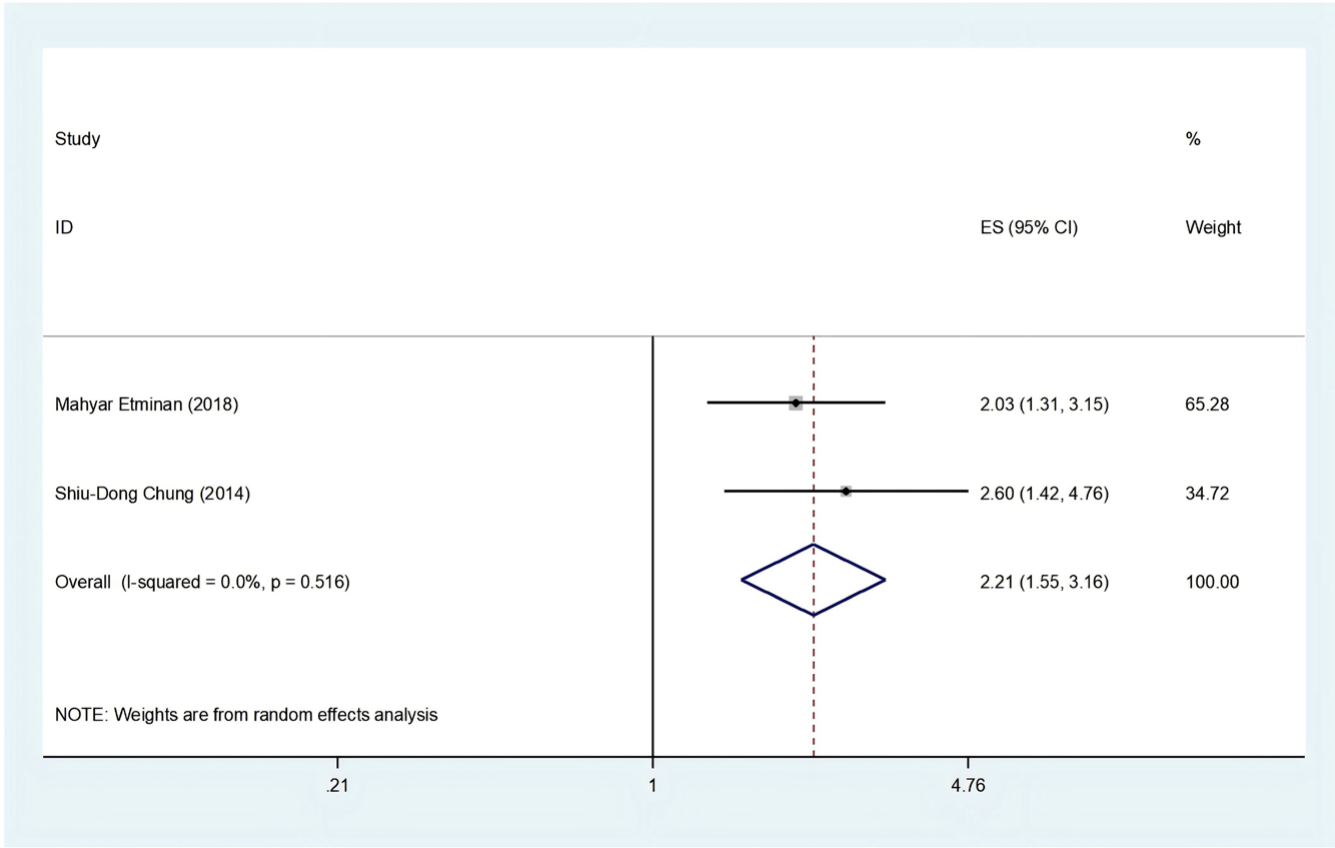
Meta-analysis forest plot of the association between nAMD and risk of PD.

In the exploration of the relationship between neovascular age-related macular degeneration (nAMD) and PD, 2 cohorts^[23,24]^ were considered. Pooled analyses revealed a significant association, indicating that individuals with nAMD faced an elevated risk of PD (HR = 2.21; 95% CI: 1.55–3.16; *I*^*2*^ = 0%, *P *< .001), and the analyses were conducted using the random-effect model. Figure 3 presents the forest plot delineating the outcomes of the meta-analysis. Sensitivity analysis given a robust result, as displayed in Figure S2, Supplemental Digital Content, http://links.lww.com/MD/N912.

### 3.6. Subgroup analysis

Subgroup analyses were conducted based on the national origin of the cohort studies. The results of these analyses revealed noteworthy associations between AMD and an elevated risk of PD. Specifically, 2 cohort studies^[21,22]^ from South Korea demonstrated a significant link, indicating that AMD is associated with an increased risk of PD (HR = 1.28; 95% CI: 1.11–1.48; *I*^*2*^ = 41.3%, *P* = .192). Two cohort studies^[12,24]^ from Taiwan, China, pooled analysis result showed that no Statistically significant evidence for this correlation (HR = 1.75; 95% CI: 0.93–3.28; *I*^*2*^ = 76.54%, *P* = .039). Furthermore, a study^[23]^ from Canada independently confirmed the connection between AMD and an elevated risk of PD (HR = 2.03; 95% CI: 1.31–3.15). The results of the subgroup analyses are displayed in Table 2.

**Table 2:** Subgroup analysis for the risk of Parkinson disease in patients with age-related macular degeneration.

Subgroups	Included studies	HR (95% CI)	Heterogeneity	
			*I*^*2*^(%)	*P*-value
Country				
Taiwan, China	2	1.75 (0.93, 3.28)	76.5	.039
South Korea	2	1.28 (1.11, 1.48)	41.3	.192
Canada	1	2.03 (1.31, 3.15)	–	–
AMD type				
Neovascular age-related macular degeneration	2	2.21 (1.55, 3.16)	0	.516
Age-related macular degeneration	5	1.44 (1.22, 1.71)	68.3	.013

### 3.7. Publication bias

**Figure 4.**
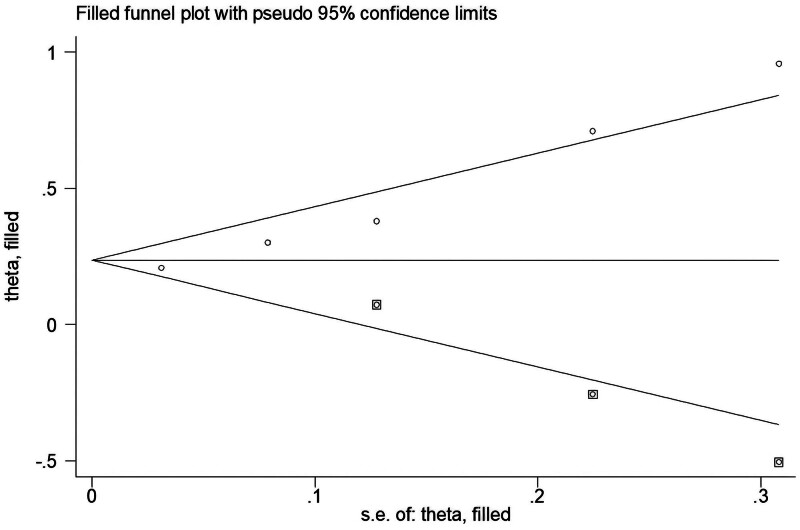
Trim and fill funnel plot of publication bias.

A visual examination of the trim and fill funnel plot reveals significant publication bias across all studies investigating the correlation between AMD and the risk of PD (Fig. 4). Subsequently, an Egger test was performed (*P* = .002, *P* < .05), providing additional confirmation of the publication bias.

## 4. Discussion

### 4.1. Main findings

The analysis comprised 5 cohort studies involving 4,771,416 individuals. The comprehensive meta-analysis revealed that both AMD and neovascular nAMD were associated with an approximately 1.44-fold and 2.21-fold increased risk of PD, respectively.

### 4.2. Interpretation of findings

To our knowledge, no prior systematic review has investigated the connection between AMD and the risk of PD. Our meta-analysis has produced noteworthy insights, revealing that individuals with both AMD and neovascular nAMD encounter an elevated risk of developing PD. The mechanisms underpinning the identified correlation between AMD and PD risk remain largely unclear, but plausible explanations include inflammation, oxidative stress, autophagy, and mitophagy.

#### 4.2.1. Inflammation

Inflammation is pivotal in AMD and PD. Multiple studies have conclusively demonstrated the presence of inflammatory mediators, including Tumor Necrosis Factor (TNF), Interleukin-1β (IL-1β), Interleukin-6 (IL-6), and Interferon-gamma (IFNγ), in the cerebrospinal fluid (CSF) and substantia nigra pars compacta (SNpc) region of the brain in PD patients.^[25–30]^ AMD studies reveal macrophage accumulation and mast cell degranulation in the choroid, fostering chronic inflammation.^[31,32]^ This widespread inflammation links both conditions. Microglia-driven neuro-inflammation notably harms AMD neurons.^[33]^ Minocycline, a tetracycline, curbs IL-6, Chemokine (C-C motif) ligand-2 (CCL2), and reactive oxygen species (ROS) in microglial cells, protecting photoreceptors.^[34]^ Early studies on 1-methyl-4-phenyl-1,2,3,6-tetrahydropyridine (MPTP)-induced PD in mice shows early substantia nigra (SN) microglial activation, leading to dopamine neuron loss.^[35]^ Additionally, Minocycline treatment post-MPTP reduces IL-1β, defends dopaminergic neurons, and mitigates microglia-related toxicity.^[36,37]^

#### 4.2.2. Oxidative stress

Oxidative stress is a pivotal factor in the pathogenesis of both AMD and PD. The brain and retina, characterized by high oxygen consumption,^[38,39]^ often generate ROS during cellular metabolism.^[40]^ While ROS play a role in normal cellular metabolism under physiological conditions,^[41]^ an imbalance in redox homeostasis occurs when ROS production exceeds antioxidant system capacity, leading to oxidative stress.^[38]^ The critical defense mechanism in regulating redox homeostasis is nuclear factor erythroid 2-related factor 2 (Nrf2).^[42,43]^ Loss or mutations in Parkinsonism associated deglycase (DJ-1)/Parkinson protein 7 (PARK7) can result in autosomal recessive early-onset PD.^[44]^ Research by Bonilha et al identified structural changes in the retina, including thinning of the retinal pigment epithelium and outer plexiform layer, in DJ-1 knockout mice. Induced selective oxidative damage through Sodium Iodate (NaIO_3_) injection in DJ-1 knockout mice accelerated retinal atrophy, with significant downregulation of Nrf2 and increased oxidative stress in retinal cells.^[45]^ This emphasizes the intricate interplay between oxidative stress and neurodegenerative processes in both AMD and PD.

#### 4.2.3. Autophagy

Moreover, autophagy also plays a crucial role in PD and AMD by actively participating in the clearance of damaged cells and proteins, essential for maintaining cellular homeostasis. However, dysregulated autophagy has been implicated in the pathogenic mechanisms of these diseases. In both PD models involving dopaminergic neurons and AMD models involving retinal pigment epithelial (RPE) cells, various autophagy-related signaling pathways have been identified, demonstrating either activation or inhibition. These pathways encompass the phosphoinositide 3-kinase/protein kinase B/mammalian target of rapamycin (mTOR) pathway,^[46,47]^ AMP-activated protein kinase/mTOR pathway,^[48,49]^ Sequestosome 1/Kelch-like ECH-associated protein 1/nuclear factor erythroid 2-related factor 2 pathway.^[50,51]^ Notably, consistent observations in these studies include increased microtubule-associated protein 1A/1B-light chain 3 conversion and upregulation of Beclin-1 expression. This underscores the intricate connection between dysregulated autophagy and the progression of both PD and AMD, further highlighting the multifaceted nature of these neurodegenerative disorders.

#### 4.2.4. Mitophagy

Lastly, the role of mitophagy in AMD and PD pathophysiology underscores its critical function in maintaining mitochondrial quality and cellular equilibrium. However, its dysregulation might be linked to the fundamental mechanisms of these 2 disorders. PTEN-induced putative kinase 1 (PINK1)/Parkinson protein 2, E3 ubiquitin protein ligase (PARKIN) proteins, crucial for mitophagy, are implicated in AMD and PD.^[52]^ Research by Huang et al found that in a Drosophila model of retinal degeneration, photoreceptors exhibit abnormal mitochondrial morphology and functional abnormalities. Notably, upregulating PINK1 and PARKIN expression showed protective effects against TRPP365-induced photoreceptor cell degeneration.^[53]^ These findings suggest shared pathogenesis and signaling pathways between AMD and PD, potentially heightening PD risk in AMD patients.

Beyond these mechanisms, multiple studies link nAMD with cardiovascular diseases,^[54–56]^ also recognized as PD risk factors. Hence, the augmented PD risk in nAMD patients might be attributed to vascular risk factors. Therefore, another reasonable explanation for the increased PD risk in patients with nAMD may be due to a high vascular risk.

#### 4.2.5. Interpretation of subgroup analysis

We conducted the Asian group analysis in accordance with the country, and divided the 5 studies included in 3 groups, namely South Korea, China and Canada. We noticed that the number of participants from 2 studies from China was large. The research by Chen incorporated more than 100,000 people, and <10,000 research differences may be the reason for the statistical heterogeneity of 2 studies. On the other hand, these 2 studies have not been comprehensive in South Korea research in the adjustment of the mixed factor of models. This may have some potentially unsatisfactory mixed bias. These reasons may cause the summary analysis of the Chinese Asian group without statistical significance. Similarly, a study from the Canadian database only adjusted the age in the mixed factors of the model. This may lead to excessive effects. Overall, of the 3 Asian groups, the summary results of the Asian group analysis from South Korea are closer to the final result of our charm analysis. At the same time, we also look forward to future research can be carried out in many countries and regions around the world, and we can consider enough mixed factors in the choice of modeling variables.

### 4.3. Limitations and future perspectives

The findings of this study carry clinical significance, suggesting that macular degeneration could act as an early indicator of PD. The potential role of AMD as a risk factor for PD prompts the need for further investigations to elucidate the impact of AMD severity and duration on PD development. Exploring whether treating AMD could reverse or ameliorate PD conditions is also essential. Additionally, our meta-analysis emphasizes the importance of incorporating macular assessments for early detection in PD patients. However, it’s crucial to acknowledge the inherent limitations of this study. The relatively limited number of studies included in this meta-analysis may impact the results of the funnel plot and publication bias assessment. Furthermore, due to the differences in the adjustment for confounding factors and demographic variables among the included studies, clinical heterogeneity is inevitable. Although we used a random-effects model in the meta-analysis to avoid the impact of heterogeneity on the summary results, our results should be interpreted with caution.

## 5. Conclusion

This systematic review and meta-analysis have identified a significant association between AMD and an elevated risk of PD. While these findings provide important insights into the potential link between AMD and PD, further research is needed to clarify the causal relationship. The results should be interpreted with caution, but they may offer a basis for exploring potential preventive and therapeutic strategies in future studies.

## Author contributions

**Conceptualization:** Mingxian Meng, Xiaoming Shen, Yanming Xie, Jiabin Wang.

**Data curation:** Jiabin Wang.

**Formal analysis:** Mingxian Meng.

**Investigation:** Mingxian Meng, Xiaoming Shen.

**Methodology:** Mingxian Meng.

**Resources:** Mingxian Meng, Junhong Liu.

**Software:** Mingxian Meng.

**Supervision:** Yanming Xie.

**Validation:** Junhong Liu.

**Writing – original draft:** Mingxian Meng.

**Writing – review & editing:** Mingxian Meng, Xiaoming Shen, Jiabin Wang, Junhong Liu.

## Supplementary Material

## References

[1] Bloem BR Okun MS Klein C. Parkinson’s disease. Lancet. 2021;397:2284–303. 33848468 10.1016/S0140-6736(21)00218-X

[2] Dorsey ER Bloem BR. The Parkinson pandemic – a call to action. JAMA Neurol. 2018;75:9–10. 29131880 10.1001/jamaneurol.2017.3299

[3] Dhiman V Trushna T Raj D Tiwari RR. Is ambient air pollution a risk factor for Parkinson’s disease? A meta-analysis of epidemiological evidence. Int J Environ Health Res. 2023;33:733–50. 35262433 10.1080/09603123.2022.2047903

[4] Breckenridge CB Berry C Chang ET Sielken RL Jr Mandel JS. Association between Parkinson’s disease and cigarette smoking, rural living, well-water consumption, farming and pesticide use: systematic review and meta-analysis. PLoS One. 2016;11:e0151841. 27055126 10.1371/journal.pone.0151841 PMC4824443

[5] Yan D Zhang Y Liu L Shi N Yan H. Pesticide exposure and risk of Parkinson’s disease: dose-response meta-analysis of observational studies. Regul Toxicol Pharmacol. 2018;96:57–63. 29729297 10.1016/j.yrtph.2018.05.005

[6] Han G Han J Han K Youn J Chung TY Lim DH. Visual acuity and development of Parkinson’s disease: a nationwide cohort study. Mov Disord. 2020;35:1532–41. 32710579 10.1002/mds.28184

[7] Flaxman SR Bourne RRA Resnikoff S. Vision Loss Expert Group of the Global Burden of Disease Study. Global causes of blindness and distance vision impairment 1990 to 2020: a systematic review and meta-analysis. Lancet Glob Health. 2017;5:e1221–34. 29032195 10.1016/S2214-109X(17)30393-5

[8] Guymer RH Campbell TG. Age-related macular degeneration. Lancet. 2023;401:1459–72. 36996856 10.1016/S0140-6736(22)02609-5

[9] Salehi MA Mohammadi S Gouravani M Rezagholi F Arevalo JF. Retinal and choroidal changes in AMD: a systematic review and meta-analysis of spectral-domain optical coherence tomography studies. Surv Ophthalmol. 2023;68:54–66. 35908660 10.1016/j.survophthal.2022.07.006

[10] Zhou WC Tao JX Li J. Optical coherence tomography measurements as potential imaging biomarkers for Parkinson’s disease: a systematic review and meta-analysis. Eur J Neurol. 2021;28:763–74. 33107159 10.1111/ene.14613

[11] Jabbehdari S Oganov AC Rezagholi F. Age-related macular degeneration and neurodegenerative disorders: shared pathways in complex interactions. Surv Ophthalmol. 2023;69:303–10. 38000700 10.1016/j.survophthal.2023.11.003

[12] Chen PJ Wan L Lai JN. Increased risk of Parkinson’s disease among patients with age-related macular degeneration. BMC Ophthalmol. 2021;21:426. 34886822 10.1186/s12886-021-02196-8 PMC8662906

[13] Page MJ McKenzie JE Bossuyt PM. The PRISMA 2020 statement: an updated guideline for reporting systematic reviews. BMJ. 2021;372:n71. 33782057 10.1136/bmj.n71 PMC8005924

[14] Taylor KS Mahtani KR Aronson JK. Summarising good practice guidelines for data extraction for systematic reviews and meta-analysis. BMJ Evid Based Med. 2021;26:88–90. 10.1136/bmjebm-2020-111651 33632720

[15] Wells G Shea B O’Connell D. Newcastle-Ottawa Quality Assessment Scale Cohort Studies. University of Ottawa. 2014.

[16] Nikolakopoulou A Mavridis D Salanti G. Demystifying fixed and random effects meta-analysis. Evid Based Ment Health. 2014;17:53–7. 24692250 10.1136/eb-2014-101795 PMC13404554

[17] Spineli LM Pandis N. Meta-analysis: random-effects model. Am J Orthod Dentofacial Orthop. 2020;157:280–2. 32005481 10.1016/j.ajodo.2019.10.007

[18] Higgins JP Thompson SG. Controlling the risk of spurious findings from meta-regression. Stat Med. 2004;23:1663–82. 15160401 10.1002/sim.1752

[19] Egger M Davey Smith G Schneider M Minder C. Bias in meta-analysis detected by a simple, graphical test. BMJ. 1997;315:629–34. 9310563 10.1136/bmj.315.7109.629 PMC2127453

[20] Schmidheiny K. A short guide to stata 14. Kurt *Schmidheiny Name/Teaching/Stataguide pdf*. 2016.

[21] Yoon JM Lim DH Youn J. Increased risk of Parkinson’s disease amongst patients with age-related macular degeneration and visual disability: a nationwide cohort study. Eur J Neurol. 2023;30:2641–9. 37243434 10.1111/ene.15896

[22] Choi S Jahng WJ Park SM Jee D. Association of age-related macular degeneration on Alzheimer or Parkinson disease: a retrospective cohort study. Am J Ophthalmol. 2020;210:41–7. 31712068 10.1016/j.ajo.2019.11.001

[23] Etminan M Samii A He B. Risk of Parkinson’s disease in patients with neovascular age-related macular degeneration. J Curr Ophthalmol. 2018;30:365–7. 30555972 10.1016/j.joco.2018.08.004 PMC6277243

[24] Chung SD Ho JD Hu CC Lin HC Sheu JJ. Increased risk of Parkinson disease following a diagnosis of neovascular age-related macular degeneration: a retrospective cohort study. Am J Ophthalmol. 2014;157:464–9.e1. 24315292 10.1016/j.ajo.2013.09.026

[25] Banati RB Daniel SE Blunt SB. Glial pathology but absence of apoptotic nigral neurons in long-standing Parkinson’s disease. Mov Disord. 1998;13:221–7. 9539333 10.1002/mds.870130205

[26] Gerhard A Pavese N Hotton G. In vivo imaging of microglial activation with [11C] (R)-PK11195 PET in idiopathic Parkinson’s disease. Neurobiol Dis. 2006;21:404–12. 16182554 10.1016/j.nbd.2005.08.002

[27] Hunot S Dugas N Faucheux B. Fcepsilon RII/CD23 is expressed in Parkinson’s disease and induces, in vitro, production of nitric oxide and tumor necrosis factor-alpha in glial cells. J Neurosci. 1999;19:3440–7. 10212304 10.1523/JNEUROSCI.19-09-03440.1999 PMC6782235

[28] McGeer PL Itagaki S Boyes BE McGeer EG. Reactive microglia are positive for HLA-DR in the substantia nigra of Parkinson’s and Alzheimer’s disease brains. Neurology. 1988;38:1285–91. 3399080 10.1212/wnl.38.8.1285

[29] Vawter MP Dillon-Carter O Tourtellotte WW Carvey P Freed WJ. TGFbeta1 and TGFbeta2 concentrations are elevated in Parkinson’s disease in ventricular cerebrospinal fluid. Exp Neurol. 1996;142:313–22. 8934562 10.1006/exnr.1996.0200

[30] Zimmermann M Brockmann K. Blood and cerebrospinal fluid biomarkers of inflammation in Parkinson’s disease. J Parkinsons Dis. 2022;12(s1):S183–200. 35661021 10.3233/JPD-223277 PMC9535573

[31] Behnke V Wolf A Langmann T. The role of lymphocytes and phagocytes in age-related macular degeneration (AMD). Cell Mol Life Sci. 2020;77:781–8. 31897541 10.1007/s00018-019-03419-4 PMC11104950

[32] Ogura S Baldeosingh R Bhutto IA. A role for mast cells in geographic atrophy. FASEB J. 2020;34:10117–31. 32525594 10.1096/fj.202000807R PMC7688488

[33] Dhodapkar RM Martell D Hafler BP. Glial-mediated neuroinflammatory mechanisms in age-related macular degeneration. Semin Immunopathol. 2022;44:673–83. 35513496 10.1007/s00281-022-00939-3

[34] Scholz R Sobotka M Caramoy A Stempfl T Moehle C Langmann T. Minocycline counter-regulates pro-inflammatory microglia responses in the retina and protects from degeneration. J Neuroinflammation. 2015;12:209. 26576678 10.1186/s12974-015-0431-4 PMC4650866

[35] Heavener KS Bradshaw EM. The Aging Immune System in Alzheimer’s and Parkinson’s Diseases. Semin Immunopathol. 2022;44:649–57. 35505128 10.1007/s00281-022-00944-6 PMC9519729

[36] Wu DC Jackson-Lewis V Vila M. Blockade of microglial activation is neuroprotective in the 1-methyl-4-phenyl-1,2,3,6-tetrahydropyridine mouse model of Parkinson disease. J Neurosci. 2002;22:1763–71. 11880505 10.1523/JNEUROSCI.22-05-01763.2002 PMC6758858

[37] Giuliani F Hader W Yong VW. Minocycline attenuates T cell and microglia activity to impair cytokine production in T cell-microglia interaction. J Leukoc Biol. 2005;78:135–43. 15817702 10.1189/jlb.0804477

[38] Beatty S Koh H Phil M Henson D Boulton M. The role of oxidative stress in the pathogenesis of age-related macular degeneration. Surv Ophthalmol. 2000;45:115–34. 11033038 10.1016/s0039-6257(00)00140-5

[39] Niu Y Zhang J Dong M. Nrf2 as a potential target for Parkinson’s disease therapy. J Mol Med (Berl). 2021;99:917–31. 33844027 10.1007/s00109-021-02071-5

[40] Bedard K Krause KH. The NOX family of ROS-generating NADPH oxidases: physiology and pathophysiology. Physiol Rev. 2007;87:245–313. 17237347 10.1152/physrev.00044.2005

[41] Ray PD Huang BW Tsuji Y. Reactive oxygen species (ROS) homeostasis and redox regulation in cellular signaling. Cell Signal. 2012;24:981–90. 22286106 10.1016/j.cellsig.2012.01.008 PMC3454471

[42] Nguyen T Nioi P Pickett CB. The Nrf2-antioxidant response element signaling pathway and its activation by oxidative stress. J Biol Chem. 2009;284:13291–5. 19182219 10.1074/jbc.R900010200 PMC2679427

[43] Rangasamy T Cho CY Thimmulappa RK. Genetic ablation of Nrf2 enhances susceptibility to cigarette smoke-induced emphysema in mice. J Clin Invest. 2004;114:1248–59. 15520857 10.1172/JCI21146 PMC524225

[44] Bonifati V Rizzu P van Baren MJ. Mutations in the DJ-1 gene associated with autosomal recessive early-onset parkinsonism. Science. 2003;299:256–9. 12446870 10.1126/science.1077209

[45] Bonilha VL Bell BA Rayborn ME. Loss of DJ-1 elicits retinal abnormalities, visual dysfunction, and increased oxidative stress in mice. Exp Eye Res. 2015;139:22–36. 26215528 10.1016/j.exer.2015.07.014 PMC4573318

[46] Li Y Wang C Liu Y You J Su G. Autophagy, lysosome dysfunction and mTOR inhibition in MNU-induced photoreceptor cell damage. Tissue Cell. 2019;61:98–108. 31759414 10.1016/j.tice.2019.09.008

[47] Rathore AS Singh SS Birla H. Curcumin modulates p62-Keap1-Nrf2-mediated autophagy in rotenone-induced Parkinson’s disease mouse models. ACS Chem Neurosci. 2023;14:1412–23. 10.1021/acschemneuro.2c00706 36989171

[48] Chen CL Chen YH Liang CM Tai MC Lu DW Chen JT. Glucosamine-induced autophagy through AMPK-mTOR pathway attenuates lipofuscin-like autofluorescence in human retinal pigment epithelial cells in vitro. Int J Mol Sci. 2018;19:1416. 29747425 10.3390/ijms19051416 PMC5983587

[49] Park JS Choe K Lee HJ Park TJ Kim MO. Neuroprotective effects of osmotin in Parkinson’s disease-associated pathology via the AdipoR1/MAPK/AMPK/mTOR signaling pathways. J Biomed Sci. 2023;30:66. 37568205 10.1186/s12929-023-00961-z PMC10422754

[50] Zhao X Wang J Li P Tang L Bai Y. Casein kinase 2-interacting protein-1 alleviates high glucose-reduced autophagy, oxidative stress, and apoptosis in retinal pigment epithelial cells via activating the p62/KEAP1/NRF2 signaling pathway. J Ophthalmol. 2021;2021:6694050. 33628480 10.1155/2021/6694050 PMC7892229

[51] Aki T Funakoshi T Unuma K Uemura K. Impairment of autophagy: from hereditary disorder to drug intoxication. Toxicology. 2013;311:205–15. 23851159 10.1016/j.tox.2013.07.001

[52] Li J Yang D Li Z. PINK1/Parkin-mediated mitophagy in neurodegenerative diseases. Ageing Res Rev. 2023;84:101817. 36503124 10.1016/j.arr.2022.101817

[53] Huang Z Ren S Jiang Y Wang T. PINK1 and Parkin cooperatively protect neurons against constitutively active TRP channel-induced retinal degeneration in Drosophila. Cell Death Dis. 2016;7:e2179. 27054334 10.1038/cddis.2016.82 PMC4855661

[54] Hyman L Schachat AP He Q Leske MC. Hypertension, cardiovascular disease, and age-related macular degeneration. Age-related macular degeneration risk factors study group. Arch Ophthalmol. 2000;118:351–8. 10721957 10.1001/archopht.118.3.351

[55] Delcourt C Michel F Colvez A Lacroux A Delage M Vernet M-H; POLA Study Group. Associations of cardiovascular disease and its risk factors with age-related macular degeneration: the POLA study. Ophthalmic Epidemiol. 2001;8:237–49. 11471092 10.1076/opep.8.4.237.1613

[56] van Leeuwen R Klaver CC Vingerling JR Hofman A de Jong PT. The risk and natural course of age-related maculopathy: follow-up at 6 1/2 years in the Rotterdam study. Arch Ophthalmol. 2003;121:519–26. 12695249 10.1001/archopht.121.4.519

