# Supplementary material for: The association between age-related macular degeneration and risk of Parkinson disease: A systematic review and meta-analysis
Source: Medicine (Baltimore). 2024 Nov 15;103(46):e40524. doi: 10.1097/MD.0000000000040524 (PMC11576027; doi:10.1097/MD.0000000000040524)

**Supplementary Table S1| PubMed database search strategy and retrieval results**

| No. | Content | Result |
| --- | --- | --- |
| #1 | Search: "Parkinson Disease"[Mesh] Sort by: Most Recent | 83,352 |
| #2 | Search: ((((((((((Idiopathic Parkinson's Disease[Title/Abstract]) OR (Lewy Body Parkinson's Disease[Title/Abstract])) OR (Parkinson's Disease, Idiopathic[Title/Abstract])) OR (Parkinson's Disease, Lewy Body[Title/Abstract])) OR (Parkinson Disease, Idiopathic[Title/Abstract])) OR (Parkinson's Disease[Title/Abstract])) OR (Idiopathic Parkinson Disease[Title/Abstract])) OR (Lewy Body Parkinson Disease[Title/Abstract])) OR (Primary Parkinsonism[Title/Abstract])) OR (Parkinsonism, Primary[Title/Abstract])) OR (Paralysis Agitans[Title/Abstract]) Sort by: Most Recent | 110,667 |
| #3 | Search: ("Parkinson Disease"[Mesh]) OR (((((((((((Idiopathic Parkinson's Disease[Title/Abstract]) OR (Lewy Body Parkinson's Disease[Title/Abstract])) OR (Parkinson's Disease, Idiopathic[Title/Abstract])) OR (Parkinson's Disease, Lewy Body[Title/Abstract])) OR (Parkinson Disease, Idiopathic[Title/Abstract])) OR (Parkinson's Disease[Title/Abstract])) OR (Idiopathic Parkinson Disease[Title/Abstract])) OR (Lewy Body Parkinson Disease[Title/Abstract])) OR (Primary Parkinsonism[Title/Abstract])) OR (Parkinsonism, Primary[Title/Abstract])) OR (Paralysis Agitans[Title/Abstract])) Sort by: Most Recent | 130,359 |
| #4 | Search: "Macular Degeneration"[Mesh] Sort by: Most Recent | 30,533 |
| #5 | Search: ((((((((((((((((((Degeneration, Macular[Title/Abstract]) OR (Macular Degenerations[Title/Abstract])) OR (Maculopathy[Title/Abstract])) OR (Maculopathies[Title/Abstract])) OR (Macular Dystrophy[Title/Abstract])) OR (Dystrophy, Macular[Title/Abstract])) OR (Macular Dystrophies[Title/Abstract])) OR (Age-Related Macular Degeneration[Title/Abstract])) OR (Age Related Macular Degeneration[Title/Abstract])) OR (Age-Related Macular Degenerations[Title/Abstract])) OR (Macular Degeneration, Age-Related[Title/Abstract])) OR (Macular Degeneration, Age Related[Title/Abstract])) OR (Maculopathies, Age-Related[Title/Abstract])) OR (Maculopathy, Age-Related[Title/Abstract])) OR (Maculopathy, Age Related[Title/Abstract])) OR (Age-Related Maculopathies[Title/Abstract])) OR (Age Related Maculopathies[Title/Abstract])) OR (Age-Related Maculopathy[Title/Abstract])) OR (Age Related Maculopathy[Title/Abstract])  Sort by: Most Recent | 29,684 |
| #6 | Search: ("Macular Degeneration"[Mesh]) OR (((((((((((((((((((Degeneration, Macular[Title/Abstract]) OR (Macular Degenerations[Title/Abstract])) OR (Maculopathy[Title/Abstract])) OR (Maculopathies[Title/Abstract])) OR (Macular Dystrophy[Title/Abstract])) OR (Dystrophy, Macular[Title/Abstract])) OR (Macular Dystrophies[Title/Abstract])) OR (Age-Related Macular Degeneration[Title/Abstract])) OR (Age Related Macular Degeneration[Title/Abstract])) OR (Age-Related Macular Degenerations[Title/Abstract])) OR (Macular Degeneration, Age-Related[Title/Abstract])) OR (Macular Degeneration, Age Related[Title/Abstract])) OR (Maculopathies, Age-Related[Title/Abstract])) OR (Maculopathy, Age-Related[Title/Abstract])) OR (Maculopathy, Age Related[Title/Abstract])) OR (Age-Related Maculopathies[Title/Abstract])) OR (Age Related Maculopathies[Title/Abstract])) OR (Age-Related Maculopathy[Title/Abstract])) OR (Age Related Maculopathy[Title/Abstract]))  Sort by: Most Recent | 43,321 |
| #7 | Search: (("Macular Degeneration"[Mesh]) OR (((((((((((((((((((Degeneration, Macular[Title/Abstract]) OR (Macular Degenerations[Title/Abstract])) OR (Maculopathy[Title/Abstract])) OR (Maculopathies[Title/Abstract])) OR (Macular Dystrophy[Title/Abstract])) OR (Dystrophy, Macular[Title/Abstract])) OR (Macular Dystrophies[Title/Abstract])) OR (Age-Related Macular Degeneration[Title/Abstract])) OR (Age Related Macular Degeneration[Title/Abstract])) OR (Age-Related Macular Degenerations[Title/Abstract])) OR (Macular Degeneration, Age-Related[Title/Abstract])) OR (Macular Degeneration, Age Related[Title/Abstract])) OR (Maculopathies, Age-Related[Title/Abstract])) OR (Maculopathy, Age-Related[Title/Abstract])) OR (Maculopathy, Age Related[Title/Abstract])) OR (Age-Related Maculopathies[Title/Abstract])) OR (Age Related Maculopathies[Title/Abstract])) OR (Age-Related Maculopathy[Title/Abstract])) OR (Age Related Maculopathy[Title/Abstract]))) AND (("Parkinson Disease"[Mesh]) OR (((((((((((Idiopathic Parkinson's Disease[Title/Abstract]) OR (Lewy Body Parkinson's Disease[Title/Abstract])) OR (Parkinson's Disease, Idiopathic[Title/Abstract])) OR (Parkinson's Disease, Lewy Body[Title/Abstract])) OR (Parkinson Disease, Idiopathic[Title/Abstract])) OR (Parkinson's Disease[Title/Abstract])) OR (Idiopathic Parkinson Disease[Title/Abstract])) OR (Lewy Body Parkinson Disease[Title/Abstract])) OR (Primary Parkinsonism[Title/Abstract])) OR (Parkinsonism, Primary[Title/Abstract])) OR (Paralysis Agitans[Title/Abstract]))) Sort by: Most Recent | 120 |

**Supplementary Table S2 | Embase database search strategy and retrieval results**

| No. | Content | Result |
| --- | --- | --- |
| #1 | macular AND ('degeneration'/exp OR degeneration) | 54,666 |
| #2 | 'degeneration, macular':ab,ti OR 'macular degenerations':ab,ti OR 'maculopathy':ab,ti OR 'maculopathies':ab,ti OR 'macular dystrophy':ab,ti OR 'dystrophy, macular':ab,ti OR 'macular dystrophies':ab,ti OR 'age-related macular degeneration':ab,ti OR 'age related macular degeneration':ab,ti OR 'age-related macular degenerations':ab,ti OR 'macular degeneration, age-related':ab,ti OR 'macular degeneration, age related':ab,ti OR 'maculopathies, age-related':ab,ti OR 'maculopathy, age-related':ab,ti OR 'maculopathy, age related':ab,ti OR 'age-related maculopathies':ab,ti OR 'age related maculopathies':ab,ti OR 'age-related maculopathy':ab,ti OR 'age related maculopathy':ab,ti | 38,969 |
| #3 | #1 OR #2 | 60,081 |
| #4 | 'parkinson disease'/exp | 198,928 |
| #5 | 'idiopathic parkinsons disease':ab,ti OR 'lewy body parkinsons disease':ab,ti OR 'parkinsons disease, idiopathic':ab,ti OR 'parkinsons disease, lewy body':ab,ti OR 'parkinson disease, idiopathic':ab,ti OR 'parkinsons disease':ab,ti OR 'idiopathic parkinson disease':ab,ti OR 'lewy body parkinson disease':ab,ti OR 'primary parkinsonism':ab,ti OR 'parkinsonism, primary':ab,ti OR 'paralysis agitans':ab,ti | 1,938 |
| #6 | #4 OR #5 | 199,223 |
| #7 | #3 AND #6 | 777 |

**Supplementary Table S3 | Cochran Library database search strategy and retrieval results**

| No. | Content | Result |
| --- | --- | --- |
| #1 | MeSH descriptor: [Macular Degeneration] explode all trees | 3,330 |
| #2 | (Degeneration, Macular OR Macular Degenerations OR Maculopathy OR Maculopathies OR Macular Dystrophy OR Dystrophy, Macular OR Macular Dystrophies OR Age-Related Macular Degeneration OR Age Related Macular Degeneration OR Age-Related Macular Degenerations OR Macular Degeneration, Age-Related OR Macular Degeneration, Age Related OR Maculopathies, Age-Related OR Maculopathy, Age-Related OR Maculopathy, Age Related OR Age-Related Maculopathies OR Age Related Maculopathies OR Age-Related Maculopathy OR Age Related Maculopathy):ti,ab,kw | 4,195 |
| #3 | #1 OR #2 | 5,627 |
| #4 | MeSH descriptor: [Parkinson Disease] explode all trees | 6,251 |
| #5 | (Idiopathic Parkinson's Disease OR Lewy Body Parkinson's Disease OR Parkinson's Disease, Idiopathic OR Parkinson's Disease, Lewy Body OR Parkinson Disease, Idiopathic OR Parkinson's Disease OR Idiopathic Parkinson Disease OR Lewy Body Parkinson Disease OR Primary Parkinsonism OR Parkinsonism, Primary OR Paralysis Agitans):ti,ab,kw | 12,508 |
| #6 | #4 OR #5 | 12,508 |
| #7 | #3 AND #6 | 7 |

**Supplementary Table S4 | Web of science database search strategy and retrieval results**

| No. | Content | Result |
| --- | --- | --- |
| #1 | TS=(Macular Degeneration OR Degeneration, Macular OR Macular Degenerations OR Maculopathy OR Maculopathies OR Macular Dystrophy OR Dystrophy, Macular OR Macular Dystrophies OR Age-Related Macular Degeneration OR Age Related Macular Degeneration OR Age-Related Macular Degenerations OR Macular Degeneration, Age-Related OR Macular Degeneration, Age Related OR Maculopathies, Age-Related OR Maculopathy, Age-Related OR Maculopathy, Age Related OR Age-Related Maculopathies OR Age Related Maculopathies OR Age-Related Maculopathy OR Age Related Maculopathy) | 49,725 |
| #2 | TS=(Parkinson Disease OR Idiopathic Parkinson's Disease OR Lewy Body Parkinson's Disease OR Parkinson's Disease, Idiopathic OR Parkinson's Disease, Lewy Body OR Parkinson Disease, Idiopathic OR Parkinson's Disease OR Idiopathic Parkinson Disease OR Lewy Body Parkinson Disease OR Primary Parkinsonism OR Parkinsonism, Primary OR Paralysis Agitans) | 159,589 |
| #3 | #1 AND #2 | 203 |

**Supplementary Table S5. The quality assessment of cohort studies.**

| Study | Year | Selection | Comparability | Outcome | Total |
| --- | --- | --- | --- | --- | --- |
| Je Moon Yoon | 2023 | *** | ** | *** | 8 |
| Po-Yu Jay Chen | 2021 | **** | ** | *** | 9 |
| Seulggie Choi | 2019 | **** | ** | *** | 9 |
| Mahyar Etminan | 2018 | **** | * | *** | 8 |
| Shiu-Dong Chung | 2014 | **** | ** | *** | 9 |

The NOS scale was used to evaluate the quality of the cohort studies.

Supplemnetary Figure S1


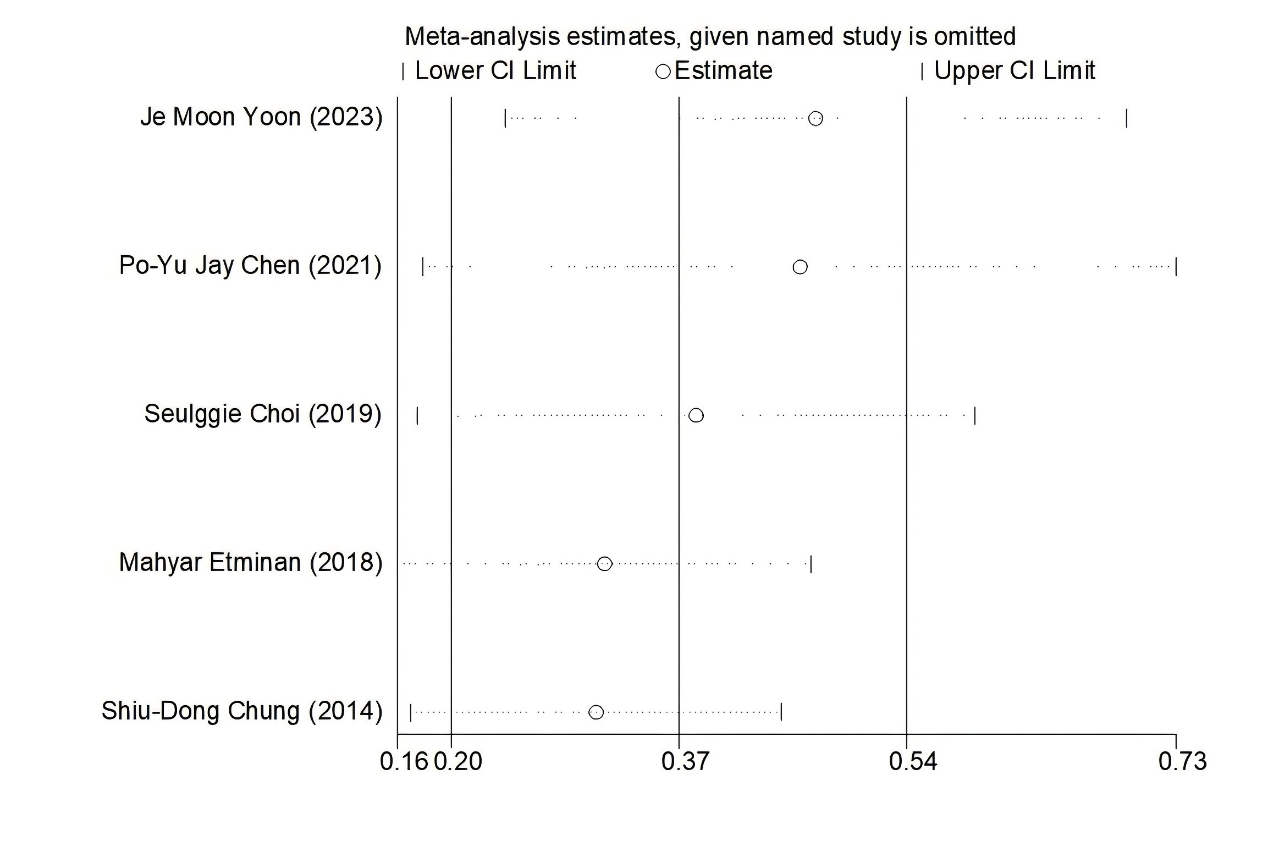


Supplemnetary Figure S2


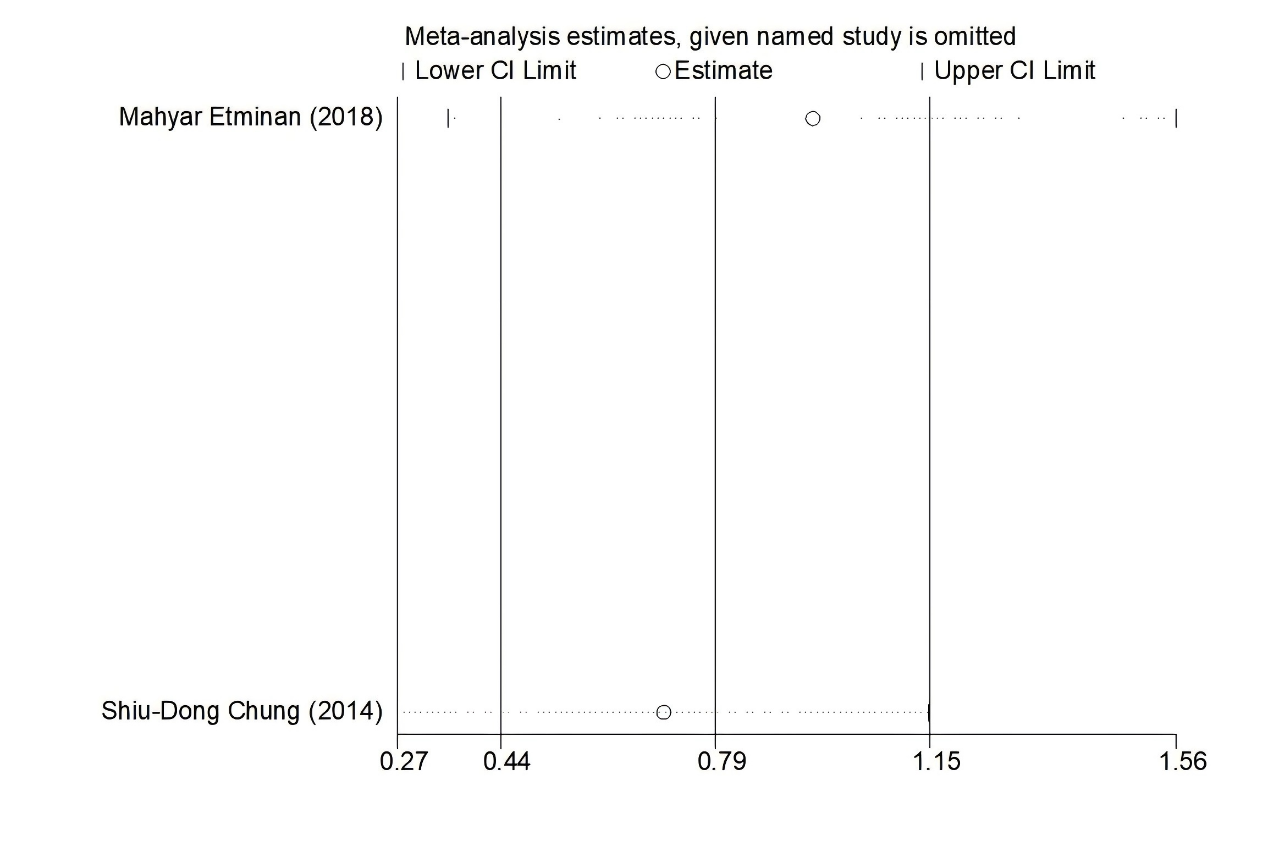

Supplement: Supplementary file 1 [file medi-103-e40524-s001.docx]
